# Signal Amplification in an Optical and Dielectric Biosensor Employing Liquid Crystal-Photopolymer Composite as the Sensing Medium

**DOI:** 10.3390/bios11030081

**Published:** 2021-03-13

**Authors:** Hassanein Shaban, Shih-Chun Yen, Mon-Juan Lee, Wei Lee

**Affiliations:** 1 Institute of Imaging and Biomedical Photonics, College of Photonics, National Yang Ming Chiao Tung University, Guiren District, Tainan 71150, Taiwan; hassanein.shaban@sci.asu.edu.eg (H.S.); loppet3@gmail.com (S.-C.Y.); 2 Department of Basic Science, Faculty of Engineering, The British University in Egypt, El Sherouk City 11837, Cairo, Egypt; 3 Department of Bioscience Technology, Chang Jung Christian University, Guiren District, Tainan 71101, Taiwan; 4 Department of Medical Science Industries, Chang Jung Christian University, Guiren District, Tainan 71101, Taiwan

**Keywords:** liquid crystal, photopolymer, UV exposure, bovine serum albumin, protein assay, dielectric spectroscopy

## Abstract

An optical and dielectric biosensor based on a liquid crystal (LC)–photopolymer composite was established in this study for the detection and quantitation of bovine serum albumin (BSA). When the nematic LC E7 was doped with 4-wt.% NOA65, a photo-curable prepolymer, and photopolymerized by UV irradiation at 20 mW/cm^2^ for 300 s, the limit of detection determined by image analysis of the LC optical texture and dielectric spectroscopic measurements was 3400 and 88 pg/mL for BSA, respectively, which were lower than those detected with E7 alone (10 μg/mL BSA). The photopolymerized NOA65, but not the prepolymer prior to UV exposure, contributed to the enhanced optical signal, and UV irradiation of pristine E7 in the absence of NOA65 had no effect on the optical texture. The effective tilt angle *θ*, calculated from the real-part dielectric constant *ε*’, decreased with increasing BSA concentration, providing strong evidence for the correlation of photopolymerized NOA65 to the intensified disruption in the vertically oriented LC molecules to enhance the optical and dielectric signals of BSA. The optical and dielectric anisotropy of LCs and the photo-curable dopant facilitate novel quantitative and signal amplification approaches to potential development of LC-based biosensors.

## 1. Introduction

Biosensors are devices designed for the detection of biologically relevant small molecules, biomolecules, biomolecular interactions such as the binding between antigen and antibody, or whole cells such as bacteria and viruses. The biological signals produced by the target of detection are transduced through the biosensor into electrical, thermal or optical signals for further qualitative and quantitative data processing. Liquid crystals (LCs) are considered novel biosensing media because of their sensitive optical response to biomolecules, thus enabling label-free bioassays based on LCs to be established. LC-based biosensing technologies can be subdivided into two platforms, one of which relies on detection at the LC-water interface, whereas the other employs the LC-glass interface.

Biosensing at the LC-water interface was applied in the detection of proteins [1], lipids [2,3], amphiphilic molecules [4,5], DNA [6], enzymatic activity [7,8] and immunocomplexes [9]. This design is characterized by the capacity for real-time detection as biological materials are water-soluble, but the optical signal derived from the LC texture can only provide qualitative or semiquantitative results. On the other hand, biodetection at the LC-glass interface is categorized as an end-point assay, where the biorecognition and biomolecular reactions are completed on a glass substrate prior to LC cell assembly with another glass substrate. In fact, sandwiching LCs between two glass substrates as an LC cell enables the external application of an electric field so that not only the optical anisotropy but also the electrical and electro-optical properties of LCs can be utilized in biosensing [10]. By exploring various LCs other than the narrow-nematic-range 5CB used in most LC-based biosensors, we developed a quantitative protein assay and immunoassay in conjunction with transmission spectrometry [11,12,13,14], as well as LC-based capacitive [15], electro-optical [14] and dielectric biosensors [16].

One of the major technical hurdles of such label-free biosensing techniques is the limited approaches for signal amplification. Gold nanoparticles were reported in several studies to enhance the optical signal of LCs by forming complexes with the target of detection or by altering the surface topology of the sensing interface to increase the extent of disturbance in the ordered alignment of LCs [17,18,19]. Our previous work demonstrated that by using LCs of high birefringence [20,21,22] or by exploiting the electrically inducible potential of LCs [23], the optical signal in LC-based biosensors can be promoted. Modification of the LC alignment layer—say, the surfactant dimethyloctadecyl[3-(trimethoxysilyl)propyl]ammonium chloride (DMOAP), a common vertical alignment reagent—coated on the glass substrate with ultraviolet (UV) irradiation [20] and adjusting the polarization direction of linearly polarized light in accordance with the rubbing direction of the glass substrate [13] also contribute to signal amplification.

Various non-LC materials, such as dichroic dyes, chiral compounds and nanomaterials, can be incorporated into LCs, giving rise to composite materials such as dye-doped LCs and cholesteric LCs that exhibit unique characteristics unattainable with pristine LCs. We previously reported that the optical signal derived from a single-substrate biosensor based on LC-photopolymer composites can be enhanced by fine-tuning the level of photopolymerization of the dopant, the photo-curable NOA65 prepolymer [24]. Studies have shown that when a mixture of NOA65 and the nematic LC E7 was exposed to UV, the vertical phase separation due to the difference in surface tension between NOA65 and E7 led to the accumulation and polymerization of NOA65 at the LC-glass interface [25,26]. The gravel-like NOA65 photopolymer thus increased the roughness and polarity of the glass surface, and was therefore exploited to control the pretilt angle of the LC molecules. When the concentration of NOA65 doped in E7 was increased from 0 wt.% to 2.5 wt.%, the pretilt angle of E7 reduced from 87.3° to 2.5° [25].

To further our understanding of the mechanism of signal amplification provided by photopolymerized NOA65, an optical and dielectric biosensing system based on the UV-cured NOA65/E7 composite was established in this study for the detection and quantitation of bovine serum albumin (BSA), a common calibration standard for the determination of protein concentrations in biological analytes. Instead of the single-substrate platform constructed in our previous work [24], the NOA65/E7 composite was sandwiched between two parallel glass substrates as a LC cell to facilitate the application of an electric field for dielectric analysis. Optical and dielectric measurement were performed on the NOA65/E7 composites at various NOA65 concentrations, UV intensities and exposure times to study the effect of the level of photopolymerization on signal amplification. The dielectric anisotropy of LCs and results derived from dielectric measurements offered a new approach to study the effect of photopolymerized NOA65 on LC orientation and signal amplification.

## 2. Experimental

### 2.1. Materials

Optical glass substrates with dimensions 22 mm × 18 mm × 1.1 mm were obtained from Ruilong Glass, Miaoli, Taiwan. Indium–tin-oxide (ITO)-coated conductive glass slides, a pair of which produces an overlapped electrode area of 5.0 mm × 5.0 mm, were manufactured by Chipset Technology Co., Ltd., Miaoli, Taiwan. The nematic LC E7 used in this study is produced by Daily Polymer Corp., Kaohsiung, Taiwan. The birefringence Δ*n* of E7 at a wavelength of 589 nm and a temperature of 20 °C is 0.2255, with the real part of the dielectric constant parallel and perpendicular to the LC molecular axis, *ε*_‖_ = 19.5 and *ε*_⊥_ = 5.2, respectively, at a frequency of 1 kHz. The photo-curable prepolymer NOA65, which is an adhesive commonly included in polymer-dispersed LCs, was obtained from Norland Products, Inc., Cranbury, NJ, USA. Vertical alignment of LCs was achieved with DMOAP, which was purchased from Sigma–Aldrich, St. Louis, MO, USA. DMOAP self-assembled into a monolayer on a glass substrate and effectively aligned LC molecules in the direction of its long alkyl chain, –CH_3_(CH_2_)_16_CH_2_. BSA, a conventional protein standard consisting of 583 amino acid residues with a molecular weight of approximately 66 kDa, was provided by Sigma–Aldrich, St. Louis, MO, USA.

### 2.2. Preparation of DMOAP-Coated Glass Substrates

**Figure 1:**
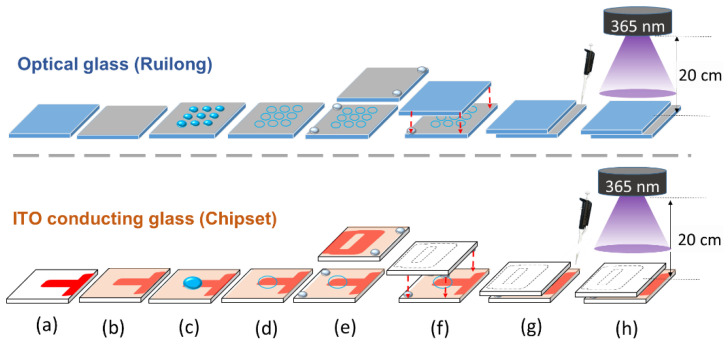
Procedures for establishing the biosensing platform based on the liquid crystal (LC)–photopolymer composite in which LC cells were assembled with either optical glass or indium–tin-oxide (ITO) conductive glass for optical or dielectric analysis, respectively. The glass substrates were cleaned to remove contaminants (**a**), followed by coating with dimethyloctadecyl[3-(trimethoxysilyl)propyl]ammonium chloride (DMOAP) as the vertical alignment layer (**b**) and immobilization with bovine serum albumin (BSA) (**c**). After drying at 35 °C on a hot plate (**d**), a mixture of 5.5 μm ball spacer and AB glue was applied at the corners of a pair of substrates (**e**) for the assembly of the LC cell (**f**). The NOA65/E7 mixture was then injected with a micropipette (**g**) and irradiated with UV light at irradiance of 5–20 mW/cm^2^ (**h**).

Steps for preparing substrates used in the cell platform for biological detection are shown in Figure 1. Optical or ITO glass slides were first cleaned by sonication in a detergent solution and then washed twice in deionized (DI) water and once in ethanol, with each procedure lasted for 15 min with sonication (Figure 1a). The substrates were dried with nitrogen and then baked in an oven at 74 °C for 30 min. Each cleaned substrate was dipped under the application of ultrasound in a 0.1% (*v*/*v*) DMOAP solution for 15 min, and then washed twice with DI water for 15 min to facilitate self-assembly of the monolayer (Figure 1b). The dip-coated substrate was blown with nitrogen and heated in the oven at 85 °C for 15 min to cure the aligning monolayer for imposing vertical alignment of LC molecules.

### 2.3. Fabrication of the LC Cell and Immobilization of BSA Molecules

BSA solutions of concentrations ranging from 10^−12^ to 10^−5^ g/mL were prepared in DI water. For BSA immobilization on a DMOAP-coated optical glass substrate, a 3 × 3 protein array was formed with 3 µL BSA solution per spot (Figure 1c). For the DMOAP-coated ITO glass substrate, a 33-µL BSA solution was dispensed to cover the entire 0.25-cm^2^ electrode area. The BSA solution on the glass substrates was dried for 20 min on a hot plate with the temperature set at 35 °C (Figure 1d). 5.5-µm ball spacers and an AB glue were mixed and dispensed on the two corners of the upper substrate (without BSA) and the lower substrate (with immobilized BSA), respectively, as shown in Figure 1e. LC cell assembly was performed by gently pressing the pair of glass substrates together and allowing the AB glue to dry for 30 min (Figure 1f). The cell gap of the assembled LC cell was measured by optical interferometry with an Ocean Optics HR2000+ high-resolution USB fiber-optic spectrometer [27]. Each LC cell was then filled with a mixture of E7 and NOA65 through capillary action by injecting the mixture with a micropipette from the side of the LC cell (Figure 1g), followed by UV exposure at wavelength of 365 nm with a Panasonic Aicure UJ35 LED Spot Type UV Curing System to induce photopolymerization (Figure 1h).

### 2.4. Optical Measurement and Image Analysis with the ImageJ Software

An Olympus BX51-P polarizing optical microscope (POM) with crossed polarizers in the transmission mode was employed for the observation of LC textures, and images with a resolution of 2048 × 1536 pixels were taken with an Olympus XC30 digital camera. To perform quantitative analysis, ImageJ, an open-source image processing and analysis program, was used to determine the relative intensity of each optical texture image by averaging the brightness of the three primary colors of RGB (0–255) with the formula V = (R + G + B)/3.

### 2.5. Dielectric Measurement

The real part of the dielectric constant was measured by a Hioki 3522-50 LCR meter, which was interfaced with a computer through a GPIB interface card and the LabVIEW graphic control program. A probe AC voltage of no more than 0.1 V was applied, which was lower than the transition threshold voltage of E7 within a frequency range of 10 Hz to 100 kHz. The real part of the dielectric spectra at various BSA concentration was recorded, and the dielectric constant at a frequency of 1 kHz was used in protein quantitative analysis.

## 3. Results and Discussion

### 3.1. Biosensing Based on the Optical Measurement of the LC–Photopolymer Composite

LC-based biosensing is facilitated by the interaction between biomolecules and LCs, whose orientation responds sensitively to the disturbance caused by the analyte, thereby generating optical and electro-optical signals that are proportional to the amount of biomolecules. The LC molecules are aligned homeotropically on a substrate coated with a vertical alignment film (DMOAP), giving rise to a dark state when observed under the POM. When biomolecules (BSA) were immobilized on the DMOAP-coated substrate, the vertical anchoring energy of DMOAP was weakened so that the LC molecules were more inclined to arrange randomly, resulting in light leakage and a dark-to-bright transition in the optical texture. In general, an enhanced optical response is considered to be correlated to an increase in the amount of the analyte located at the LC-glass interface. To improve the sensitivity and limit of detection, a photo-curable prepolymer NOA65 was added to the nematic E7 in this study to further amplify the optical signal.

**Figure 2:**
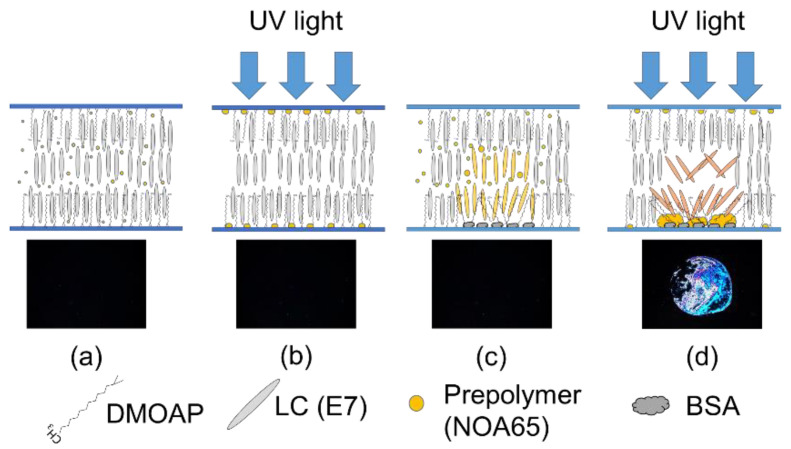
The working principle of the biosensing platform based on the LC–photopolymer composite. (**a**) Before UV exposure and in the absence of biomolecules, the LC molecules are vertically aligned in the presence of a minute concentration of NOA65, resulting in a completely dark optical texture. (**b**) After UV exposure, polymer gravels of NOA65 form on the substrates but are still unable to weaken the vertical anchoring of DMOAP. (**c**) In the presence of immobilized biomolecules at relatively low concentrations, the optical texture is still dark, which implies that the tilt angle of the LC molecules was not significantly affected. (**d**) To achieve signal amplification, the mixture of LC and NOA65 was irradiated with UV to induce the polymerization of NOA65, which leads to significant change in the tilt angle of LC molecules, giving rise to enhanced brightness in the optical texture.

As shown in Figure 2a, in the absence of analytes the vertical anchoring strength of DMOAP was unaffected when a minute concentration of the NOA65 prepolymer was dispersed in E7. After UV exposure, the NOA65 prepolymer phase-separated and aggregated as small polymer gravels on the glass substrate due to the difference in surface tension between NOA65 and E7 [25,26], but the tilt angle of LCs remained unchanged (Figure 2b). In the presence of a trace number of biomolecules, the orientation of LCs was slightly disturbed and the alignment effect of DMOAP was masked by the analyte, but because of the strong vertical anchoring exerted by DMOAP from both glass substrates, optical signals derived from light leakage was undetectable (Figure 2c). Nevertheless, when photopolymerization of NOA65 was induced by UV exposure, surface roughness on the glass substrate was higher in the area with the immobilized biomolecules than that with DMOAP modification only, leading to greater disruption in LC orientation and, in turn, an enhanced optical signal (Figure 2d). It is assumed that the ionic and polar side chains of amino acids distributed on the surface of a protein analyte (BSA in this study) attracted the relatively hydrophilic NOA65, which coalesced around BSA while being repelled by the hydrophobic alkyl chain of DMOAP, bringing about the difference in surface roughness in a situation similar to the preparation of self-positioning NOA65 micro lens [28]. In addition, light scattering can be attributed to refractive-index mismatch in the multi-regional boundaries between the LC molecules, photopolymerized NOA65, DMOAP, BSA, and the glass substrate. As a consequence, LC molecules in the proximity of BSA were disturbed to a greater extent compared with those in direct contact with DMOAP, contributing to signal amplification of BSA without simultaneously increasing the background.

#### 3.1.1. Experimental Conditions for the Preparation of the LC–Photopolymer Composite

**Figure 3:**
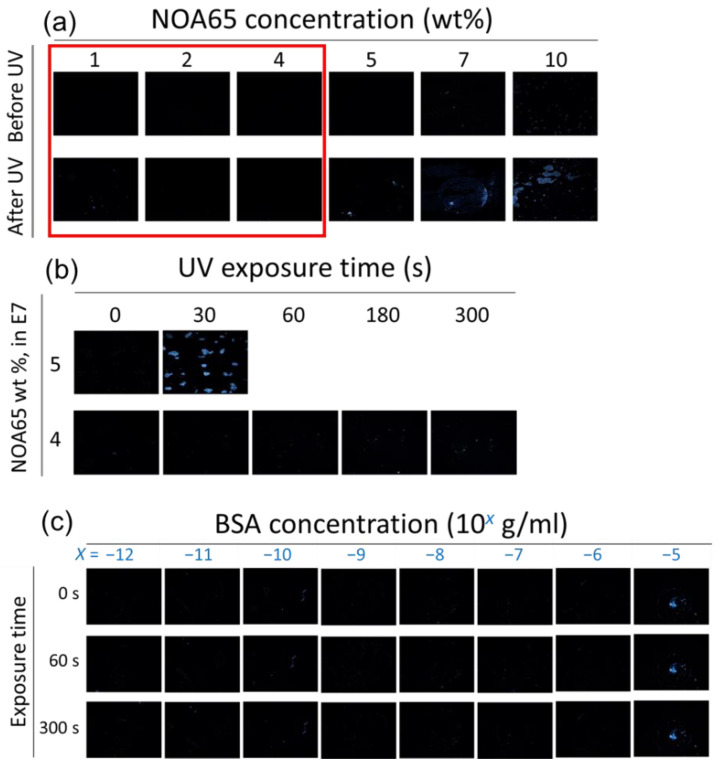
The optical texture of the LC–photopolymer composites at various NOA65 concentrations and UV exposure times. (**a**) E7 was doped with 1, 2, 4, 5, 7, or 10 wt.% NOA65 followed by exposure to 10-mW/cm^2^ UV light for 30 s. (**b**) E7 was doped with 4 or 5 wt.% NOA65 followed by exposure to 20-mW/cm^2^ UV light for 0, 30, 60, 180, or 300 s. (**c**) The optical texture of pristine E7 in the presence of immobilized BSA molecules. BSA was immobilized at concentrations ranging from 10^−12^ to 10^−5^ g/mL, followed by LC cell assembly and UV irradiation at 20 mW/cm^2^ for 0, 60, or 300 s.

To avoid false-positive optical signals, the extent of photopolymerization of NOA65 was carefully controlled so that in the absence of BSA the LC molecules remained vertically anchored and a dark background was observed for the LC–photopolymer composite under the POM with crossed polarizers. As a protein-free reference, DI water instead of BSA was used as the sample, which was dispensed and dried on the glass substrate, followed by LC cell assembly and interaction with the mixture of E7 and NOA65, as described in Section 2.3 and Figure 1. As shown in the upper panels of Figure 3a, when E7 was doped with 1, 2, 4, 5, 7 or 10 wt.% of NOA65, the optical texture of the NOA65/E7 mixture remained dark. After irradiated with UV at 10 mW/cm^2^ for 30 s, light leakage was observed at 5-, 7- and 10-wt.% NOA65 (lower panels, Figure 3a). NOA65 concentration was thus maintained at 4 wt.% or lower in the following studies to avoid such nonspecific background noise. We then increased the UV intensity to 20 mW/cm^2^ and prolonged the exposure time to 300 s for E7 doped with 4 or 5 wt.% of NOA65 (Figure 3b). A pronounced light leakage was detected when the NOA65/E7 mixture containing 5-wt.% NOA65 was irradiated with UV for 30 s, whereas at 4 wt.% NOA65 the optical texture was completely dark up to a UV exposure time of 300 s. It can be concluded from the above results that in the absence of analytes at the LC-glass interface, LCs remained homeotropically aligned when NOA65 of concentrations ≤4 wt.% was exposed to a UV irradiance of no more than 20 mW/cm^2^ for a period of time ≤300 s.

#### 3.1.2. Protein Detection and Quantitation with the LC–Photopolymer Composite

**Figure 4:**
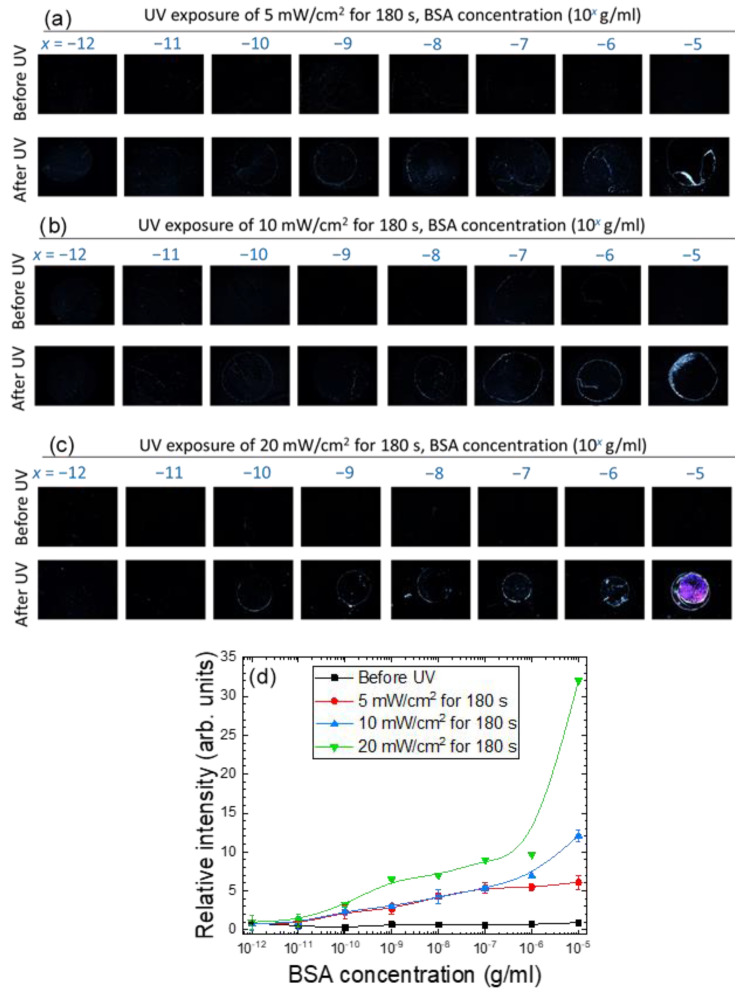
The optical texture of E7 doped with 1-wt.% NOA65 in the presence of immobilized BSA molecules at concentrations ranging from 10^−12^ to 10^−5^ g/mL, followed by LC cell assembly and UV irradiation at (**a**) 5, (**b**) 10 or (**c**) 20 mW/cm^2^ for 180 s. The brightness of the optical textures in (**a**–**c**) was quantitated with ImageJ and plotted against the BSA concentration in (**d**). Error bars represent standard deviations calculated from the relative intensities of at least three independent experiments.

To demonstrate signal amplification by photopolymerized NOA65, detection of BSA with pristine E7 as well as the NOA65/E7 composite was compared. As shown in Figure 3c, LC cells were assembled with immobilized BSA in the concentration range of 10^−12^ to 10^−5^ g/mL on one of the DMOAP-coated glass substrates, followed by injection of only E7 and UV irradiation at 20 mW/cm^2^ for 0, 60 or 300 s. Without NOA65 the lowest BSA concentration that can be discerned was 10^−5^ g/mL from the optical texture of E7 under the POM. The optical signal at each BSA concentration remained unchanged when irradiated with UV for 60 or 300 s, suggesting that exposure to UV had no effect on the orientation of E7 itself. On the other hand, when doped with 1-wt.% NOA65, the optical texture of the NOA65/E7 mixture in the presence of BSA before UV irradiation was completely dark, similar to that of pristine E7 (Figure 3c). When the LC cell was exposed to UV for 180 s at an intensity of 5, 10 or 20 mW/cm^2^, optical signals can be observed at BSA concentrations lower than 10^−5^ g/mL, and the brightness of the optical texture increased with increasing amount of BSA as well as UV intensity (Figure 4a–c), suggesting that the increase in surface roughness caused by photopolymerization of NOA65, as explained in Figure 2, was responsible for the enhanced light leakage and amplified optical signal. In order to quantitatively analyze the optical signal in relation to BSA concentration, the freeware ImageJ was used to perform image analysis and calculate the relative brightness of the optical texture. Results from the quantitative analysis, presented as a plot of relative intensity versus BSA concentration in Figure 4d, was consistent with the texture observations (Figure 4a–c). It was found that the higher the BSA concentration, the greater the enhancement in the texture brightness of the NOA65/E7 composite with UV intensities. When exposed to 20-mW/cm^2^ UV, the texture brightness of the NOA65/E7 composite was significantly higher than that exposed to 5- or 10-mW/cm^2^ UV in the higher BSA concentration range of 10^−10^–10^−5^ g/mL, but not at the lower 10^−12^ and 10^−11^ g/mL BSA concentrations. The limit of detection (LOD) was calculated according to the following equation:

(1) $$\mathrm{LOD}=\frac{3s}{m}$$

where *s* represents the standard deviation of the relative intensity (texture brightness) at the lowest BSA concentration, with its value significantly higher than that at 0-g/mL BSA, and *m* represents the slope of the linear regression [29]. Based on the results in Figure 4d, the LOD values thus obtained were 1.0 × 10^−8^, 8.8 × 10^−8^ and 6.1 × 10^−9^ g/mL BSA, when the NOA65/E7 mixture was exposed to UV irradiation of 5, 10 and 20 mW/cm^2^, respectively. The calculated LOD was consistent with the BSA concentration at which the dark-to-bright transition was observed in Figure 4a–c.

**Figure 5:**
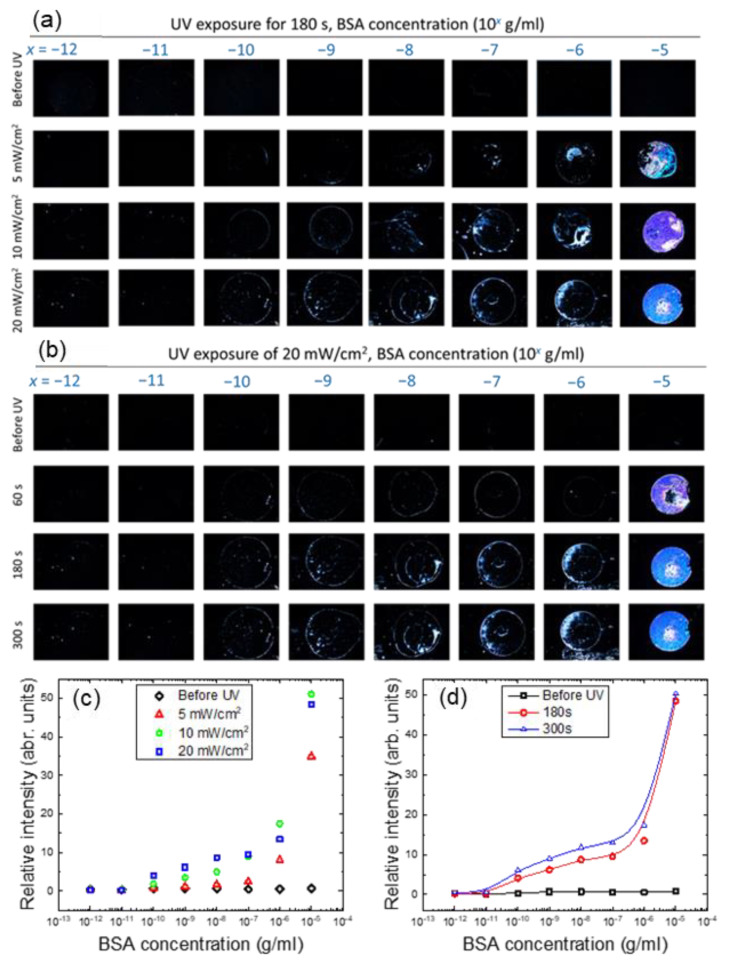
The optical texture of E7 doped with 4-wt.% NOA65 in the presence of BSA molecules. BSA was immobilized at concentrations ranging from 10^−12^ to 10^−5^ g/mL, followed by LC cell assembly and UV irradiation (**a**) at 5, 10, or 20 mW/cm^2^ for 180 s, or (**b**) at 20 mW/cm^2^ for 0, 60, 180, or 300 s. The brightness of the optical textures in Figure (**a**,**b**) was quantitated with ImageJ and plotted against the BSA concentration as depicted in Figure (**c**,**d**), respectively.

We next increased the doping concentration of NOA65 to 4 wt.%, the maximal concentration at which the homeotropic alignment of E7 can still be maintained without being significantly interfered by the photopolymerized NOA65 (Figure 3). The NOA65/E7 mixture was exposed to UV at an irradiance of 5, 10 or 20 mW/cm^2^ for 180 s (Figure 5a) or for 60, 180 or 300 s at a fixed UV irradiance of 20 mW/cm^2^ (Figure 5b). As expected, the relative intensity of the optical texture of the NOA65/E7 composite increased with increasing BSA concentration, and was further enhanced by increasing UV irradiance and exposure time, except for those at 10^−5^-g/mL BSA, where the brightness of the optical texture seemed to reach saturation and no longer correlated to UV irradiance (Figure 5c,d). The LOD values for the detection by E7 doped with 4 wt.% NOA65 and exposed to UV at 20 mW/cm^2^ for 180 and 300 s were 4.3 × 10^−8^ and 3.4 × 10^−9^ g/mL BSA, respectively. Compared with 1-wt.% NOA65, the optical signal was significantly amplified by doping E7 with 4-wt.% NOA65, especially at higher concentrations (10^−8^ to 10^−5^ g/mL) of BSA (compare Figure 4d and Figure 5c). However, at a fixed NOA65 concentration, increasing UV irradiance or prolonging UV exposure rendered relatively limited signal amplification (Figure 4d and Figure 5c,d).

In one of our previous studies, a single-substrate biodetection platform was constructed by spin-coating a thin layer of the NOA65/E7 composite film on a DMOAP-modified glass substrate, thus eliminating the procedure for LC cell assembly [24]. Since the thickness of the LC thin film (4.5 ± 0.5 μm) was less than the cell gap of the LC cell (5.5 ± 0.5 µm) assembled in this study, and the vertical anchoring strength at the LC-air interface was much weaker in comparison with that imposed by DMOAP at the LC-glass interface, it is predictable that the LC thin film layered on a single glass substrate is more sensitive to the disturbance caused by biomolecules. The LOD determined for the single-substrate detection was 1.8 × 10^−9^ g/mL of BSA probed with a NOA65/E7 composite film containing 3 wt.% of NOA65 photopolymerized by UV irradiation at 10 mW/cm^2^ for 30 s [24]. Although assembly of an LC cell increased the complexity of the biosensing procedure, the LC film sandwiched between two parallel glass substrates was more stable and uniform in thickness. Besides, by increasing the NOA65 concentration to 4 wt.% and UV exposure to 20 mW/cm^2^ for 300 s, a similar LOD of 3.4 × 10^−9^ g/mL BSA can be achieved with the cell-based biosensing platform.

### 3.2. Biosensing Based on the Dielectric Measurement of the LC–Photopolymer Composite

**Figure 6:**
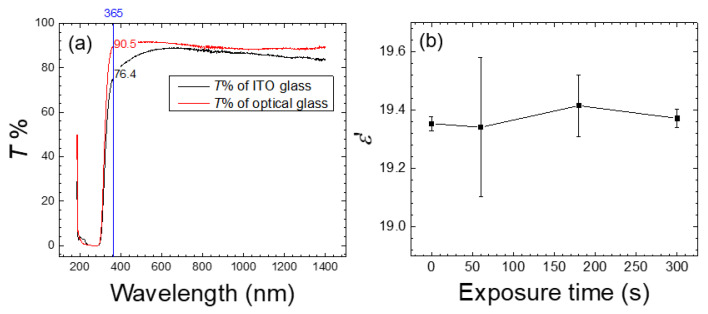
Transmission spectrometric analysis of glass substrates and dielectric measurement of E7 doped with 4-wt.% NOA65 in the absence of BSA. (**a**) Comparison of the optical transmission spectra of the optical and ITO-coated glass slides measured within a wavelength range of 200–1500 nm. (**b**) The real-part dielectric constant *ε*’ determined at a UV irradiance of 20 mW/cm^2^ with various exposure times. Error bars represent the standard deviation calculated from *ε*’ of at least three independent experiments.

For dielectric measurements, the LC cell was assembled with a pair of conductive ITO glass substrates instead of the optical glass used in optical measurements (Figure 1). As a comparison, the transmittance of ITO-coated glass substrates was lower than that of optical flat glass at 365 nm, which is the central wavelength of the UV source for photoinduced polymerization (Figure 6a). LC cells assembled with the ITO-coated glass substrates allowed for an electric field to be applied to measure the capacitance of the NOA65/E7 composite at various BSA concentrations, from which the real part of the dielectric constant was derived. The effective dielectric constant depends on the average tilt angle *θ* (measured from the substrate plane) of the LC molecules,

(2) $$\varepsilon_{\mathrm{eff}}=\varepsilon_{∥}\sin^{2}\theta +\varepsilon_{⊥}\cos^{2}\theta$$

where *ε*_eff_ represents the measured real-part dielectric constant *ε*’, and *ε*_‖_ and *ε*_⊥_ are the parallel and perpendicular components of *ε*’, respectively [15]. It is reasoned that the immobilized BSA may disturb the ordered alignment of LCs, thus altering their tilt angle and consequently the measured dielectric constant. Because surface roughness was increased due to the accumulation of the photopolymerized NOA65 at the LC-glass interface in the presence of BSA (Figure 2d), it is expected that the average tilt angle may change further so that signal amplification contributed by polymerized NOA65 aggregates can be detected and quantitated through dielectric spectroscopy. When E7 was doped with 4-wt.% NAO65 and irradiated with UV at 20 mW/cm^2^ for 0, 60, 180 or 300 s in the absence of BSA, *ε*’ remained unchanged irrespective of exposure times (Figure 6b). The slight decrease in *ε*’ at each exposure time compared with the parallel component of the dielectric constant of E7 (*ε*_‖_ *=* 19.5), which represents the state where the LC molecules were vertically aligned, may be partially attributed to the doped NOA65, which has a dielectric constant of 4.6 [30].

**Figure 7:**
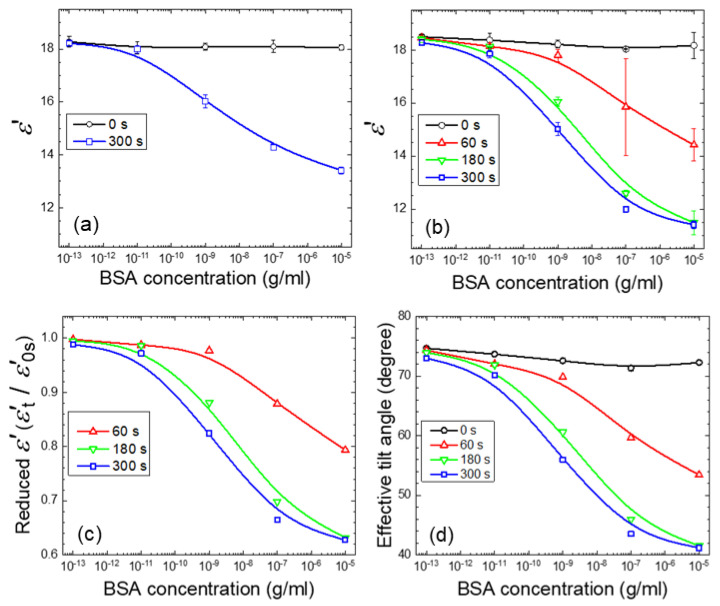
Dielectric spectroscopic analysis of NOA65/E7 cells exposed to 20-mW/cm^2^ UV irradiation in the presence of BSA. (**a**) The real-part dielectric constant of E7 doped with 2-wt.% NOA65 and exposed to UV for 0 or 300 s. (**b**) The real-part dielectric constant, (**c**) the reduced *ε*’, and (**d**) the effective tilt angle *θ* of E7 doped with 4-wt.% NOA65 and exposed to UV for 0, 60, 180, or 300 s as a function of the BSA concentration. The value of *θ* was calculated by Equation (3) based on the data retrieved from (**b**). Curves are based on spline fitting.

At a doping concentration of 2-wt.% NOA65, the NOA65/E7 composite prepared by exposure to UV at 20 mW/cm^2^ for 300 s exhibited a decrease in *ε*’ with increasing BSA concentrations, whereas *ε*’ was kept constant prior to UV irradiation (exposure time 0 s) (Figure 7a). When NOA65 concentration was increased to 4 wt.%, a similar inverse correlation to that at 2-wt.% NOA65 between *ε*’ and BSA concentration was observed (Figure 7b). At each BSA concentration, prolonged UV exposure (Figure 7b) as well as an increase in NOA65 (compare Figure 7a,b at 300 s) resulted in lower *ε*’. The value of *ε*’ decreased from 18.1 to 11.2 with increasing BSA concentration in the range of 10^−13^ to 10^−5^ g/mL at a NOA65 concentration of 4 wt.% and UV exposure of 20 mW/cm^2^ for 300 s. These results confirm our findings in optical measurements and provide strong evidence that photopolymerized NOA65 enhanced the optical and dielectric signals in LC-based biosensing. The calculated LOD for the NOA65/E7 composite at 2-wt.% and 4-wt.% NOA65 with UV exposure at 20 mW/cm^2^ for 300 s was 2.9 × 10^−10^ and 8.8 × 10^−11^-g/mL BSA, respectively. When E7 doped with 4-wt.% NOA65 and exposed to 20 mW/cm^2^ for 300 s was used as the sensing medium, the LOD determined by optical measurements was 3.4 × 10^−9^ g/mL for BSA, which was an order of magnitude higher than that obtained by dielectric measurements (8.8 × 10^−11^-g/mL BSA). In our previously reported dielectric protein assay based on dual-frequency LC (DFLC), *ε*’ at 100 kHz in the high-frequency regime increased from 3.74 ± 0.02 to 5.10 ± 0.04 while that at 200 Hz in the low-frequency regime decreased from 9.83 ± 0.04 to 8.46 ± 0.05 when BSA concentration was increased from 10^−7^ to 10^−2^ g/mL [16]. Although the absolute value of *ε*’ varied with the type of LCs and the frequency at which *ε*’ was measured, it was observed that the LOD was lowered by an order of magnitude (from 10^−6^ to 10^−7^ g/mL for BSA) compared with that determined by optical texture observation when BSA was quantitatively analyzed by DFLC-based dielectric measurements [16], which was in agreement with the findings of this study. Results from this and our previous studies therefore imply that dielectric spectroscopic analysis offers more sensitive detection of biomolecules in comparison with the qualitative or semi-quantitative optical measurements.

Calculated as *ε*’*_t_*/*ε*’_0_, where *ε*’*_t_* and *ε*’_0_ stand for *ε*’ obtained at a UV exposure time *t* and 0 s, respectively, Figure 7c displays the reduced *ε*’ against the BSA concentration deduced from Figure 7b. Expressing the dielectric signal using reduced *ε*’ enabled the measured *ε*’*_t_* under different experimental conditions to be normalized to a constant *ε*’_0_, which represents the dielectric constant of the NOA65/E7 mixture prior to UV irradiation. As a result, the maximum value of reduced *ε*’ is unity, corresponding to the unperturbed homeotropic state as in the absence of the analyte, whereas the minimum value is *ε*_⊥_/*ε*’_0_ = 5.2/19.5 = 0.267, reflecting a state where the anchoring effect of DMOAP diminished due to the accumulated analyte at the LC-glass interface. To demonstrate signal amplification by photopolymerized NOA65 more explicitly, the effective tilt angle *θ* in radians expressed by

(3) $$\theta =\sin^{-1}\sqrt{\frac{\varepsilon_{\mathrm{eff}}-\varepsilon_{⊥}}{\varepsilon_{∥}-\varepsilon_{⊥}}}=\sin^{-1}\sqrt{\frac{\varepsilon^{\prime }-5.2}{14.3}}$$

in accordance with Equation (2), was calculated for each *ε*’ in Figure 7b. As shown in Figure 7d, *θ* decreased with increasing BSA concentration and UV exposure time, suggesting the nontrivial effect of NOA65 photopolymerization on the orientation of LC molecules.

**Figure 8:**
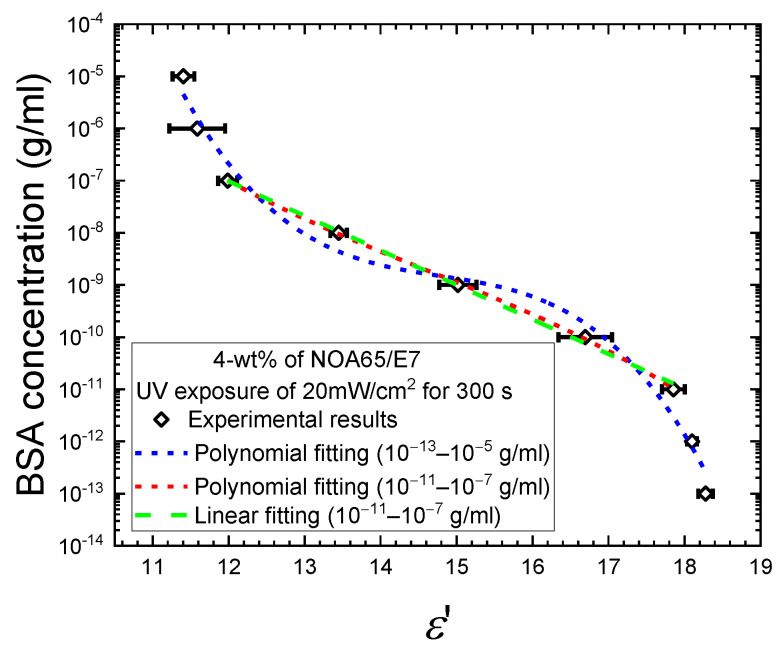
The BSA concentration as a function of the measured dielectric value, allowing interpolation of the concentration of the protein analyte in the dielectric permittivity range between 11.4 and 18.3. The blue and red dashed curves are third-order polynomial functions describing the relation of *ε’* to BSA concentration in the wider 10^−13^–10^−5^ g/mL and the narrower 10^−11^–10^−7^ g/mL range, respectively. The green dashed line represents the result of linear regression in the BSA concentration range of 10^−11^–10^−7^ g/mL.

To mathematically describe the correlation between the BSA concentration *c* and the real-part dielectric constant *ε*’, additional dielectric measurements were taken from supplementary samples with BSA concentrations at 10^−12^, 10^−10^, 10^−8^, and 10^−6^ g/m and the results were combined with the data shown in Figure 7b for the exposure time of 300 s. Figure 8 shows the complete set of the nine experimental data points and two curves fitted to polynomials of the third order with the coefficient of determination *R*^2^ = 0.9830 to cover the entire BSA concentration range of 10^−13^–10^−5^ g/mL or *R*^2^ = 0.9997 for the narrower range of 10^−11^–10^−7^ g/mL. This permits one to obtain the BSA concentration (in g/mL) simply from the measured *ε*’ value using the following equation:

(4) $$\log\left(c\right)=B_{0}+B_{1}\varepsilon \prime +B_{2}\varepsilon \prime^{2}+B_{3}\varepsilon \prime^{3}$$

where the coefficients are displayed in Table 1. The polynomial curve for the wider range of BSA concentration (10^−13^–10^−5^ g/mL) in Figure 8 can be considered as consisting of three segments, with higher slopes at the two extremes compared to the intermediate segment. In the low BSA concentration range of 10^−13^–10^−11^ g/mL, the measured *ε’* values decreased from 18.2 to 17.9, but were still close to the *ε’_‖_* value of 19.5, suggesting that the vertical anchoring strength of DMOAP dominated the control of the LC tilt angles, and the disturbance in the homeotropic alignment of LCs caused by the analyte was relatively weak. When BSA concentration was increased to the range of 10^−11^–10^−7^ g/mL, *ε’* decreased further from 17.9 to 12, which indicates that the amount of BSA reached a critical value to mask the alignment effect of DMOAP, and the decrease in dielectric signal was predominantly determined by and proportional to the amount of immobilized BSA. At high BSA concentrations of 10^−7^–10^−5^ g/mL, the extent of decrease in *ε’* (from 12 to 11.4) was again diminished. This can be explained by the saturation of BSA adsorbed on the DMOAP-coated glass surface. Increasing the amount of BSA would not give rise to proportional decrease in *ε’* as observed in the 10^−11^–10^−7^ g/mL concentration range, because excess BSA that can no longer adsorb to the DMOAP-coated glass surface was washed away during the sample preparation process. Therefore, by eliminating the *ε’* data in the BSA concentration ranges of 10^−13^–10^−11^ and 10^−7^–10^−5^ g/mL during curve fitting, the third-order polynomial fitting curve (red dashed curve, Figure 8) coincides with the linear regression line (green dashed line, Figure 8) between 10^−11^ and 10^−7^ g/mL BSA. The monotonic correlation thus revealed between *ε’* and BSA concentration supports the above explanation on the dominant effect of the amount of BSA on *ε’* in the 10^−11^–10^−7^ g/mL concentration range.

To further simplify the mathematical expression [15], linear regression using the method of least squares was performed on the *c*–*θ* curve for the UV exposure time of 300 s in Figure 7d. To improve the accuracy of linear regression analysis, the *ε*’ and *θ* values for additional BSA samples of 10^−12^, 10^−10^, 10^−8^, and 10^−6^ g/mL were measured and calculated, respectively (data not shown). When expressed by the following linear correlation,

(5) θ=alog(c)+b

where *a* stands for the slope and *b* the vertical intercept of the linear function *θ* (*c*), a calibration curve was obtained from which the concentration of an unknown protein sample can be interpolated with its *θ* value. Note that Equation (5) is apparently invalid for *c* = 0 and, to be conservative, it is limited to the experimental range of 10^−13^ g/mL ≤ *c* ≤ 10^−5^ g/mL. Such linear and inverse correlation between *θ* and *c* was also derived in our previous work on a capacitive biosensor based on a LC of high birefringence to describe the variation of *θ* over a BSA concentration range of 10^−9^ to 10^−3^ g/mL [15]. As presented in Table 2, the *R*-squared value for the regression analysis was 0.974 for the entire BSA concentration range of 10^−13^–10^−5^ g/mL. Because in most biochemical assays the range of protein concentration in an analyte usually spans only two to three orders of magnitude, two narrower ranges of linearity, 10^−11^–10^−7^ and 10^−10^–10^−8^ g/mL BSA, were selected for comparison (Table 2). By reducing the concentration range for linear fitting, the *R*-squared value and thus the accuracy of protein quantitation can be increased.

## 4. Conclusions

An optical and dielectric protein biosensor based on a LC–photopolymer composite was established in this study. Compared to our previously reported single-substrate detection, the sensing platform constructed with LC cells enabled the generation of a uniform electric field between two parallel conducting glass surfaces for dielectric spectroscopic measurements, which is a crucial advantage as biosensing in conjunction with dielectric spectroscopy led to improved sensitivity and LOD. Through optical texture observation and image analysis, it was demonstrated that by synthesizing a LC–photopolymer composite consisting of E7 impregnated with 4-wt.% NOA65, followed by UV irradiation at 20 mW/cm^2^ for 300 s, significant signal amplification was achieved for the detection of BSA. The photopolymerized NOA65, but not the prepolymer prior to UV exposure, contributed to the enhanced optical signal, and UV irradiation had no effect on the brightness of the optical texture of pristine E7 in the absence of NOA65. By subjecting the LC–photopolymer composite to an externally applied electric field, dielectric spectroscopic analysis was performed to improve the sensitivity and LOD (88 pg/mL BSA, determined by dielectric measurements), offering a novel means of quantitative protein assay. Investigating the BSA concentration dependence of the real-part dielectric constant of the LC composite led to the calculation of the effective tilt angle, which significantly decreased only when NOA65 was photopolymerized by UV. These findings strongly support that photopolymerized NOA65 altered the LC orientation to enhance the transduced optical and dielectric signals of BSA. The optical and dielectric biosensing technique based on the NOA65/E7 composite is a label-free end-point assay, which can be easily adopted to a wide variety of biochemical and clinical assays such as immunoassays and enzyme assays that rely on biomolecular interactions on a solid substrate.

## Figures and Tables

**Table 1:** Polynomial coefficients (in g/mL) in Equation (4) obtained through curve fitting of the experimental data as given in Figure 8.

*c* Range (g/mL)	*B*_0_	*B*_1_	*B*_2_	*B*_3_	*R* ^2^
10^−13^–10^−5^	238.07437 ± 69.99535	−49.61891 ± 14.72656	3.33539 ± 1.01777	−0.07501 ± 0.02311	0.98304
10^−11^–10^−7^ (polynomial)	32.18531 ± 15.9708	−7.23333 ± 3.30443	0.45545 ± 0.22561	−0.0104 ± 0.00508	0.99965
10^−11^–10^−7^ (linear)	0.93695 ± 0.25101	−0.66235 ± 0.01734	0	0	0.99795

**Table 2:** Linear regression parameters for the plot of effective tilt angle *θ* (in degrees) versus BSA concentration *c* (in g/mL). Regression analysis was performed on the *θ* values within three different BSA concentration ranges for comparison.

*c* (g/mL)	*a* (°)	*b* (°)	*R* ^2^
10^−13^–10^−5^	−4.97422	11.7882	0.97422
10^−11^–10^−7^	−6.43209	−1.74649	0.99863
10^−10^–10^−8^	−6.55982	−3.09482	0.99985

## Data Availability

The authors confirm that the data supporting the findings of this study are available within the article.
